# Fluorofenidone inhibits the epithelial-mesenchymal transition in human lens epithelial cell line FHL 124: a promising therapeutic strategy against posterior capsular opacification

**DOI:** 10.5935/0004-2749.20210088

**Published:** 2021

**Authors:** Michael Wormstone

**Affiliations:** 1 School of Biological Sciences, University of East Anglia, Norwich, United Kingdom

Dear Editor:

I have recently read an article in your journal by Zhuang et al.^(1)^ and became interested in this work, specifically because as indicated in the title, the lens cell line FHL124 was used as an experimental model. I was curious to read in the Methods section that the authors obtained these cells from American Type Culture Collection (ATCC). As far as I am aware, FHL124 cells are not commercially available. Upon accessing the ATCC website and searching for lens cells, only human lens epithelial (HLE-B3) are available for sale. Indeed, the latter are virally transformed, whereas FHL124 cells are spontaneously transformed and better reflect observations in tissue culture models^(2)^. Therefore, the authors should confirm that the cells they have used are indeed FHL124 and not HLE-B3 and, if so, clearly present the source of their FHL124 cells.

Kind regards,
